# Simultaneous Simulations of Uptake in Plants and Leaching to Groundwater of Cadmium and Lead for Arable Land Amended with Compost or Farmyard Manure

**DOI:** 10.1371/journal.pone.0047002

**Published:** 2012-10-04

**Authors:** Charlotte N. Legind, Arno Rein, Jeanne Serre, Violaine Brochier, Claire-Sophie Haudin, Philippe Cambier, Sabine Houot, Stefan Trapp

**Affiliations:** 1 Department of Environmental Engineering, Technical University of Denmark, Lyngby, Denmark; 2 Veolia Environnement – Research and Innovation, Rueil-Malmaison, France; 3 INRA, UMR 1091 Environment and Arable Crop Research Unit, Thiverval-Grignon, France; Argonne National Laboratory, United States of America

## Abstract

The water budget of soil, the uptake in plants and the leaching to groundwater of cadmium (Cd) and lead (Pb) were simulated simultaneously using a physiological plant uptake model and a tipping buckets water and solute transport model for soil. Simulations were compared to results from a ten-year experimental field study, where four organic amendments were applied every second year. Predicted concentrations slightly decreased (Cd) or stagnated (Pb) in control soils, but increased in amended soils by about 10% (Cd) and 6% to 18% (Pb). Estimated plant uptake was lower in amended plots, due to an increase of *K_d_* (dry soil to water partition coefficient). Predicted concentrations in plants were close to measured levels in plant residues (straw), but higher than measured concentrations in grains. Initially, Pb was mainly predicted to deposit from air into plants (82% in 1998); the next years, uptake from soil became dominating (30% from air in 2006), because of decreasing levels in air. For Cd, predicted uptake from air into plants was negligible (1–5%).

## Introduction

Amending soils with compost or sewage sludge is beneficial to the soil fertility due to the high content of organic matter and positive effects on the release of nutrients [1]. On the other hand, amendments may contain various metals and organic micro pollutants that could induce some potential adverse effects to terrestrial ecosystems and human health. A recent review [2] that compared municipal solid waste composts (MSW) to sewage sludge in terms of heavy metal availability in amended soils concluded that the application to soil of both types of amendments in the long run increase the total concentration of several metals in soils. However, the metal availability in compost amended soils tends to be decreased and of less risk to humans concerning exposure through the food chain, whereas amending soils with digested sludge can increase the metal availability.

The QualiAgro long-term field experiment on agronomic effects and environmental impacts of amending various composts on soil and crops has been started in September 1998 at Feucherolles, France (about 30 km west of Paris). Amendments included urban composts (biowaste compost, BIOW; municipal solid waste compost, MSW; co-compost of green waste and sewage sludge, GWS) as well as farm yard manure (FYM) and applications were compared to controls without amendment (CTR) [3].

Factors affecting uptake of heavy metals into vegetation are type of metal, plant species and cultivar, plant-related parameters such as transpiration and growth, and soil parameters like pH, organic matter, soil texture and redox status [4]. Metals that are available to the plant in the soil solution can be taken up and this fraction is often assessed from mild extractions of soil. However, robust tools for predicting the transfers of metals from soil and air to plants are scarce and often error prone due to the large variability of metal uptake in plants [5]. For Cd and Pb, most regressions for predicting plant uptake from soil correlate the concentration in the plant with soil parameters like pH, organic matter content and total metal concentration in soil e.g. [4], [6]. These are the same parameters that are applied for estimating the solubility of these metals in soil water [7]. This indicates that the water soluble fraction in soil is important for plant uptake and that dissolved metal species are transported together with the water into plants.

Plants also change the water balance of the soil: about 2/3rd of the precipitated water is transpired in most ecosystems [8]. In summer, evapotranspiration is typically higher than precipitation, and the soil dries out. Hereby, also leaching of water and solute to groundwater is reduced or stopped. On the other hand, water and solute uptake into vegetation also depends on the distribution and availability of both in soil. Consequently, water balance, solute transport, leaching to groundwater and plant uptake of solute and water are coupled processes. Recently, a coupled plant and groundwater transport model for NaCl could simulate the transpiration-induced changes in groundwater salinity [9]. However, for metals, no models that simultaneously predict plant uptake as well as leaching to groundwater were found.

The objective of this work is to present and test a model framework for the simulation of the coupled transport of water and dissolved heavy metals, the uptake of both into crops, and leaching of solute and water to groundwater. It is hypothesized that uptake of Cd and Pb from soil can be simplified as a passive uptake with soil water only. The model is dynamic and iterative and can be run for a variable number of periods (*n*). The same superposition principle as for the dynamic plant uptake model for organic compounds [10], [11] was applied, where changes in emission and input between periods were considered by superposition of the results of *n* periods. This model for uptake into plants was coupled with a tipping buckets soil water model [12], which calculates the water budget, solute transport and root uptake in the vadose zone. The model is parameterized with data derived from the ten-year field study and tested versus measured concentrations of lead (Pb) and cadmium (Cd) in soil and plants [3]. The accuracy of the model predictions can thus be evaluated. Furthermore, the simulation results will also be used to interpret the measured data.

## Results

### Measured *K_d_*’s versus Regression *K_d_*’s

**Figure 1:**
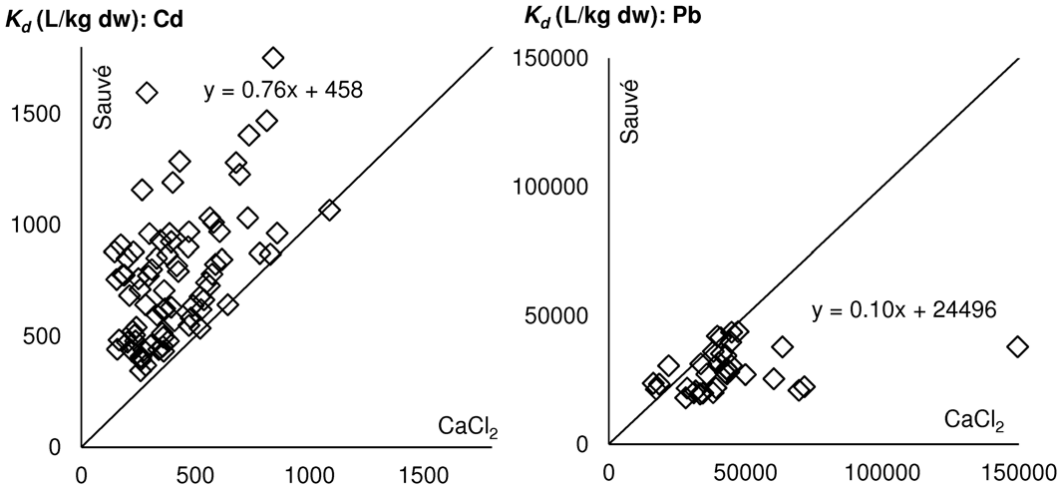
Estimated soil-water partition coefficient *K_d_* (Sauvé regression) vs. measured *K_d_* (determined from CaCl_2_ extractions). The dotted line indicates a ratio of one.

The *K_d_* estimates based on the regression equations of Sauvé et al. [7] were compared to measured *K_d_* values based on CaCl_2_ extractions of the soil surface horizons from 2002 to 2007 (Figure 1). The median ratios between predicted and measured *K_d_*’s are 1.9 (1.1; 4.4) for Cd and 0.68 (0.31; 1.3) for Pb (values in brackets are the 5^th^ and 95^th^ percentiles). Only predicted *K_d_*-values were applied for the modeling of metal adsorption in all horizons and for the whole period. The predicted *K_d_*-values for Cd in the control soil surface layer decreased over the ten-years period from 609 to 423 L kg^−1^. For the GWS plot, they were first decreasing, but the final *K_d_* was the same as the initial (588 L kg^−1^). On the contrary, the predicted *K_d_*-values for Cd in the FYM plot were increasing (from 538 to 858 L kg^−1^), as for BIOW (785 to 1437 L kg^−1^) and MSW (507 to 965 L kg^−1^). The same tendency – decreasing predicted *K_d_*-values for control and GWS plots and increasing *K_d_* for the FYM, BIOW and MSW plots, was observed for Pb. These variations are mainly related to variations of soil pH, and of organic carbon in the case of Cd (equations 23–24; Table S1).

### Results for Top Soil

**Figure 2:**
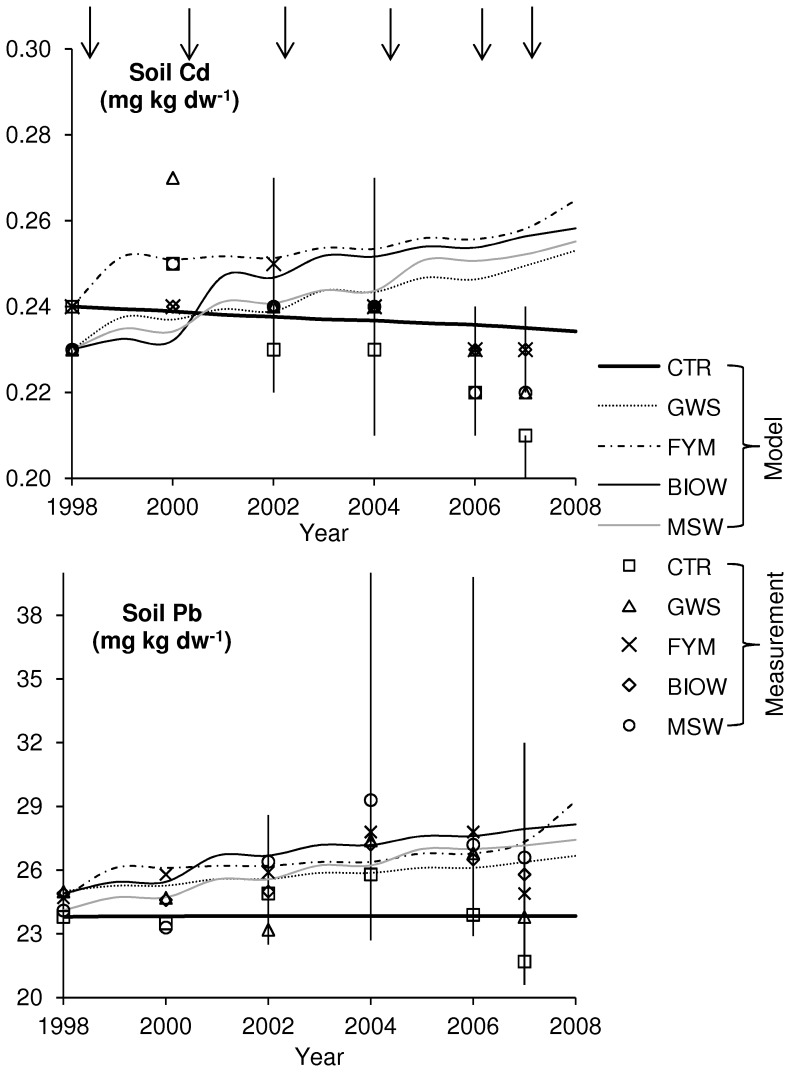
Comparison of predicted and measured concentrations in top soil for the five treatments. September 1998 to July 2007. Model predictions are connected by lines for clearer comparison to measured values. Vertical lines denote the range of measured values and arrows the time of amendment application. Measured concentrations represent median of four replicates.

Simulated and measured concentrations of Cd and Pb in top soil are shown in Figure 2. The differences between the five treatments are generally rather small. Measured values of Cd range between 0.21 and 0.27 mg kg dw^−1^ (median of four replicates), with seeming random variations versus time for the amended plots, and a decreasing trend for the control (Figure 2). Predicted concentrations of the control plot decline from 0.24 to 0.234 mg kg dw^−1^ (−2.6%), showing the slightly negative balance between the estimated air input and outputs by leaching and plant uptake.

Predicted concentrations for amended plots display non monotonous curves, related to the successive inputs from amendments and seasons dominated by outputs; however they increase overall after 10 years, GWS by 9.8%, FYM, BIOW and MSW by 10.1%, 12.1% and 10.8%, respectively. Therefore, deviations between predicted and measured concentrations of Cd occur towards the end of the simulation period, the predicted values becoming about 10% higher than the measured ones.

The predicted concentrations of Pb in the top soil of the control plot are almost constant (+0.2%) over the ten years (Figure 2). Predicted concentrations from the other plots all increase after 10 years, between 6.7% (GWS) and 18% (FYM). The medians of measured data follow this trend with the highest value being found in 2006 for all treatments and the control. However, the relative dispersion of data and unexplained drops of Pb contents toward the last year weaken the possible links between measured and simulated variations.

The modeled fluxes from top soil are presented for one simulation event with growth of maize in Table 1 for the control and the treatment with the highest input of metal by amendment (BIOW, Table 2). Similar deposition values from air were measured by Azimi et al. [13] in 2002 at Versailles, about 20 km from the study site (0.05 mg Cd m^−2^ year^−1^ and 2.20 mg Pb m^−2^ year^−1^ compared to our estimates of 0.03 and 1.97, respectively). Table 1 also shows that the predicted plant uptake is 22% (Cd) and 10% (Pb) lower for the amended soil compared to the control soil.

**Table 1:** Modeled fluxes for top soil, CTR and BIOW treatments (August 2000–October 2001).

1^st^ soil layer (mg m^−2^)	CTR		BIOW	
	Cd	Pb	Cd	Pb
m_Soil, initial_	91.4	9121	88.8	9741
Amendment	0	0	+6	+470
Air	+0.03	+1.97	+0.03	+1.97
Leaching	−0.25	−0.57	−0.21	−0.53
Plant uptake	−0.09	−0.20	−0.07	−0.18
ΔSoil	−0.31	+1.20	+5.75	+471.3

**Table 2:** Amendment application (second half of September in each given year) and input of Cd and Pb with amendment for the different treatments.

Year	Amendment application (kg dw m^−2^)				Cd Input (mg m^−2^)				Pb Input (mg m^−2^)			
	GWS	FYM	BIOW	MSW	GWS	FYM	BIOW	MSW	GWS	FYM	BIOW	MSW
1998	1.07	1.31	1.62	1.00	3.1	4.7	1.1	2.1	90	527	198	224
2000	1.98	1.10	2.45	1.92	1.3	0.6	6.0	2.9	117	36	470	324
2002	1.85	1.56	2.58	0.95	2.1	1.1	2.1	1.3	110	69	190	250
2004	1.73	1.37	1.97	1.46	1.5	1.1	1.0	2.9	91	151	160	295
2006	1.77	1.49	1.94	1.00	1.5	1.7	1.1	0.7	104	210	125	65
2007	1.58	1.33	1.62	1.05	1.6	2.7	0.8	1.3	113	730	84	101

GWS: Co-compost of green waste and sewage sludge, BIOW: Biowaste compost, FYM: Farmyard manure and MSW: Municipal solid waste compost.

### Model Results for Plants

**Figure 3:**
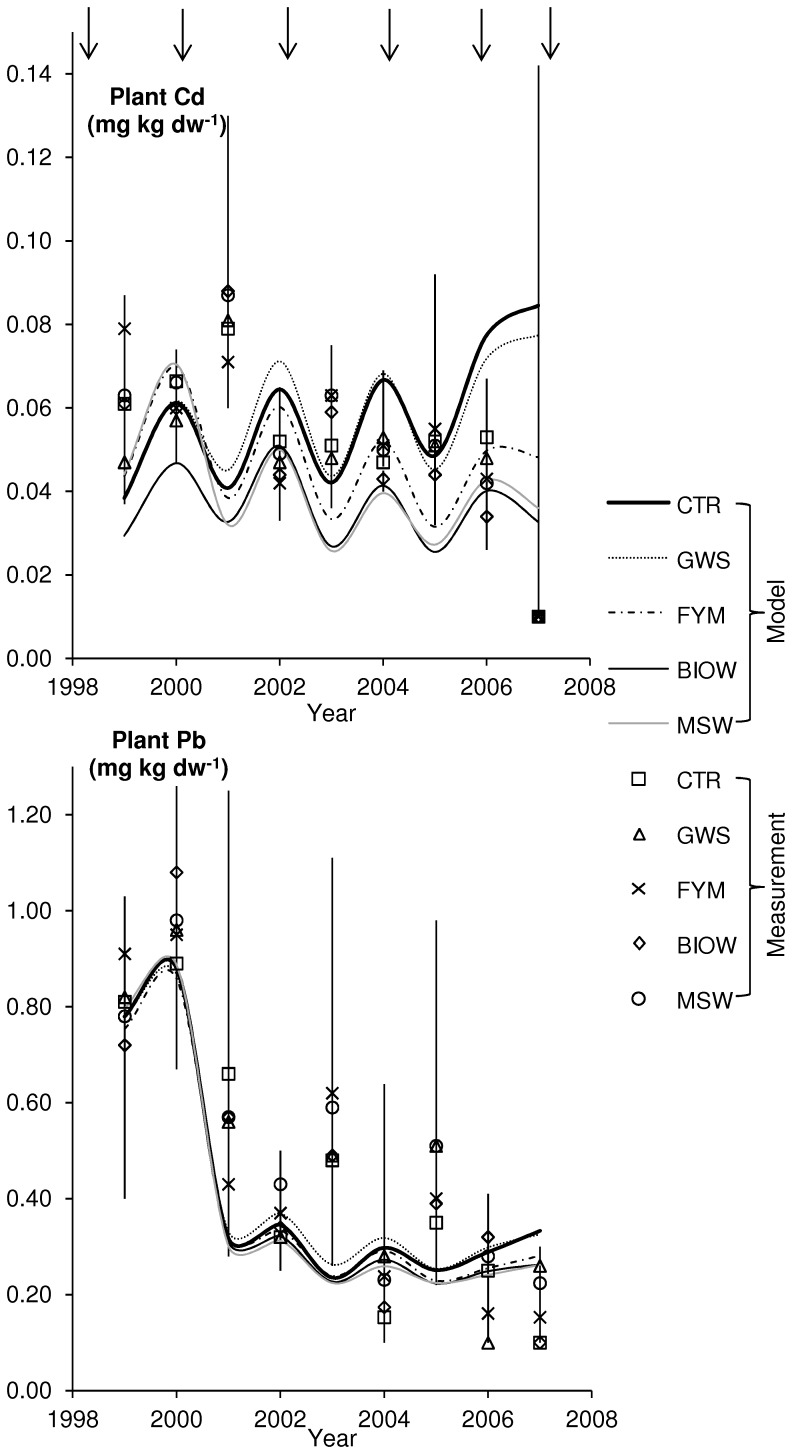
Comparison of predicted and measured concentrations in plants (mg kg dw^−1^) for the five treatments. October 1999 to July 2007. Model predictions are connected by lines for clearer comparison to measured values. Vertical lines denote the range of measured values and symbols the medians of the four replicates (values below QL were set equal to ½ QL (note that QLs from 1999–2005 were applied for all years). Top arrows recall the time of amendment application.

Simulated and measured concentrations of Cd and Pb in plants are shown in Figure 3. The predicted results for Cd are near the measured concentrations for harvest residues (leaves and stems). The measured concentrations of Cd in grains are lower than simulated and not shown in the figure, since most values were below the limit of quantification. The measured concentrations of Cd in stem and leaves are particularly low for the year 2007, which may be related to the exceptional crop barley and to a temporary change of method this year (see Chemical analyses). Before 2007, the simulated values are lower every second year, because concentrations for maize (1999, 2001, 2003 and 2005) are predicted to be lower than concentrations for wheat. The reason is that the model takes in account that maize is a C4-plant and has a lower transpiration coefficient (Table 3), i.e. less uptake of water per produced biomass [8]. The measured values for plant residues do not show this trend, the opposite is the case: the highest concentrations were measured 2001, for maize. Assuming no difference between the years, the measured concentration of Cd and Pb is significantly (p<0.01) higher in maize residues. It is known that Cd concentrations are lower in maize grain compared to wheat grain [14], but only a few studies allow comparison between wheat and maize residues. Lavado et al. [15] found for both residues and grains higher Cd concentrations in wheat compared to maize and the opposite for Pb.

Initially, the FYM and MSW plot (the curves overlap) have the highest simulated Cd concentration in plants, and the BIOW plot the lowest. The simulated concentrations of the MSW and FYM scenario decrease with time, those for the control (CTR) and GWS scenario increase. This shows that the total concentration in soil is less important for the predicted concentration in plants than the *K*_d_. According to the simulations, deposition of Cd from air is of minor relevance. Only 1 to 5% of the simulated Cd in plants stems from atmospheric deposition. Also, the harvested plant mass has only little influence on the predicted concentration. Overall, the predicted concentrations of Cd in plants are rather similar for the five plots, and do depend only marginally on the Cd applied with amendment. This is confirmed by the measured concentrations of Cd in plants: Only in three instances, maize FYM 1999 (higher), wheat GWS 2000 (lower) and wheat BIOW 2006 (lower) was the measured concentration of Cd in leaf and stem significantly (p<0.05) different for treatment and control.

Both the simulations and the measured results show a clear decreasing trend for Pb in plants in the period between 1998 and 2007 (Figure 3). According to the model simulations, the reason for the decline is the declining deposition of Pb from air. Measured concentrations of Pb in air in Paris (which served as input data for the simulation) decrease from 0.21 µg m^−3^ in 1998 to 0.01 µg m^−3^ in 2007 [16]. Subsequently, deposition from air declines, too. The fraction of Pb uptake from air into plants falls from 82% in 1998/9 (year 1) to about 30% from 2003/4 (year 6). For most of the years, the simulated Pb concentration in plants lies within the range of measured concentrations for stems or leaves. Again measured concentrations in grains are not shown, since most were below the limit of quantification. As before for Cd, the model predicts higher concentrations in wheat as in maize. The measured data are only for the first years (as long as aerial deposition dominates) higher for wheat. For the first years, as long as plants take up Pb mainly from air, there is little or no difference between the five plots. This is confirmed by the statistical analysis of the measured concentrations: only in three events (wheat BIOW 2000, higher, barley GWS and MSW 2007, higher) was the concentration of Pb in leaf and stem significantly (p<0.05) different between treatment and control. Thus, for most events, no significant difference in concentration of plants from amended and control treatments could be found. Towards the end, the control scenario (CTR) and the GWS have slightly higher simulated concentrations of Pb. Again, this is due to the decreasing *K*_d_ of these plots. The input of amendment, which was highest for the FYM and the BIOW treatment (Table 2), did not lead to elevated concentrations in plants, but to a predicted decrease, because pH and thus the *K*_d_ increased.

**Table 3:** Estimated plant parameters (initial plant mass normalized to an area of 1 m^2^).

Parameter	Symbol	Unit	Wheat	Maize	Barley	Source
Fraction of attached soil	*SA*	g ww g fw^−1^	0.001	0.001	0.001	[37]
Transpiration coefficient	*T_C_*	L kg fw^−1^	100	60	as wheat	[8]
Overall growth rate	*k_G,O_*	d^−1^	0.094	0.081	as wheat	Estimated [11]
Initial plant mass	*M_Initial_*	kg fw^−1^	0.031	as wheat	as wheat	[11]

### Leaching to Groundwater

**Figure 4:**
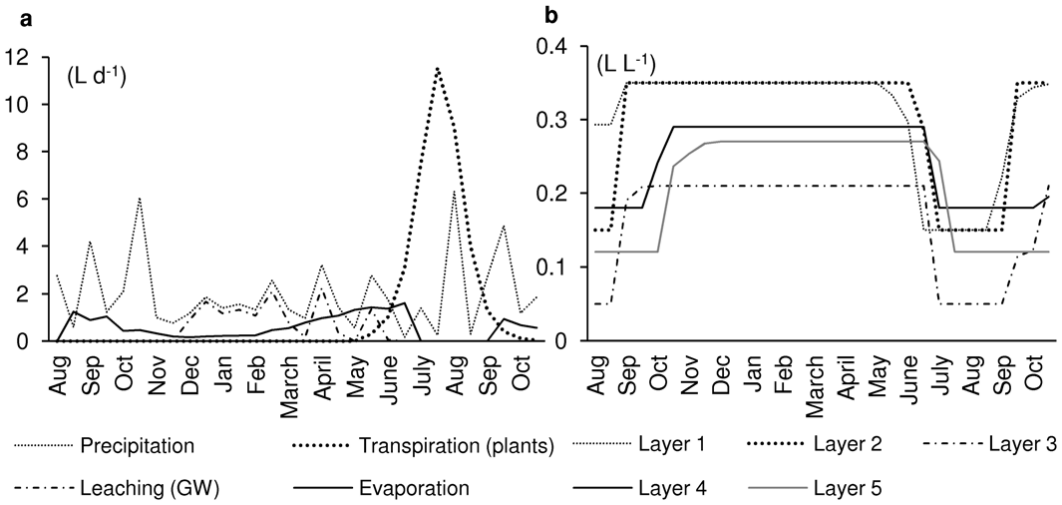
Simulated water balance and content of soil. (a) Simulated annual water balance, control scenario, August 1998 to October 1999; (b) simulated water content of the five soil layers, same simulation event.

An annual water balance for the control scenario and the period from August 1998 to October 1999 is shown in Figure 4a. The precipitation is rather equally distributed over the whole season. Transpiration of plants (maize) occurs only during the vegetation period (from May to October). Evaporation from soil is relevant from March to June, then it stops, due to the drying of the upper soil, and continues when the plants are ripening and do not take up water anymore, after September. Leaching from the lowest soil layer to groundwater takes place in winter (December to March), and in periods with elevated precipitation (April, May). The simulation of the water content of the five soil layers (Figure 4b) starts with empty soil, i.e., the initial water content is set to the permanent wilting point (which differs for the five soil layers, see Table 4). In autumn, the layers are filled up again with water, due to precipitation and low or no transpiration of the vegetation, beginning with the top layer and then downwards. The water content remains at field capacity until the vegetation starts to draw larger amounts of water for transpiration. From May, the water storage of the soil is depleted, again starting with the top layer and then downwards. In July, all five soil layers have reached the permanent wilting point. From end of August, when the plants reduce their transpiration and ripen, the water content of the soil layers increases again, starting with the top layers (Figure 4b).

**Table 4:** Measured soil parameters (depth, dry density *ρ_S,dry_*, field capacity, *FC,* and permanent wilting point, *PWP*) of the soil layers.

Soil layer	Depth	*ρ_S,dry_*	*FC*	*PWP*
	(cm)	(kg L^−1^)	(L L^−1^)	(L L^−1^)
1	0–29	1.32	0.35	0.15
2	29–35	1.53	0.35	0.15
3	35–50	1.46	0.21	0.05
4	50–90	1.50	0.29	0.18
5	90–150	1.45	0.27	0.12

The leaching of Cd and Pb is closely coupled to the leaching of water. In fact, the pattern of leaching is identical for both compounds, only the level is different. Like water, leaching of compounds occurs in the winter time, and in periods with heavy precipitation and thus water surplus. Table 5 shows the leaching of water (L m^−2^), Cd (mg m^−2^) and Pb (mg m^−2^) over the ten-year simulation for the control scenario. The annual leaching of water is very variable; the range is from 0 to 457 L m^−2^. To consider is that the lengths of the simulation periods are not equal, due to different vegetation periods of maize and wheat. The average leaching of water is 157 L water m^−2^ per year, which is 23% of the average precipitation (Table S2). The average leaching of metals is 0.07 mg Cd m^−2^ year^−1^ and 0.16 mg Pb m^−2^ year^−1^ (Table 5).

**Table 5:** Leaching of water (L m^−2^), Cd (mg m^−2^) and Pb (mg m^−2^) for the ten simulation events in the control scenario.

Period	(L m^−2^)	Cd (mg m^−2^)	Pb (mg m^−2^)
Aug 98–Oct 99	199	0.078	0.181
Nov 99–Jul 00	113	0.044	0.103
Aug 00–Oct 01	457	0.192	0.431
Nov 01–Jul 02	124	0.052	0.117
Aug 02–Oct 03	229	0.102	0.216
Nov 03–Jul 04	0.0	0.000	0.000
Aug 04–Oct 05	125	0.065	0.130
Nov 05–Jul 06	0.0	0.000	0.000
Aug 06–Jul 07	86	0.049	0.093
Aug 07–Jul 08	236	0.134	0.286
**Total**	**1568**	**0.715**	**1.557**
Average	157	0.072	0.156

According to the simulations, heavy metals applied in top soil via the various amendments do not affect the leaching of these metals, because neither Cd nor Pb are transported from top soil to bottom soil within the considered ten years. Thus, the amounts of Cd and Pb that leach to groundwater do not depend on the type of amendment. Some differences are seen because of the different initial concentrations of the five plots.

**Figure 5:**
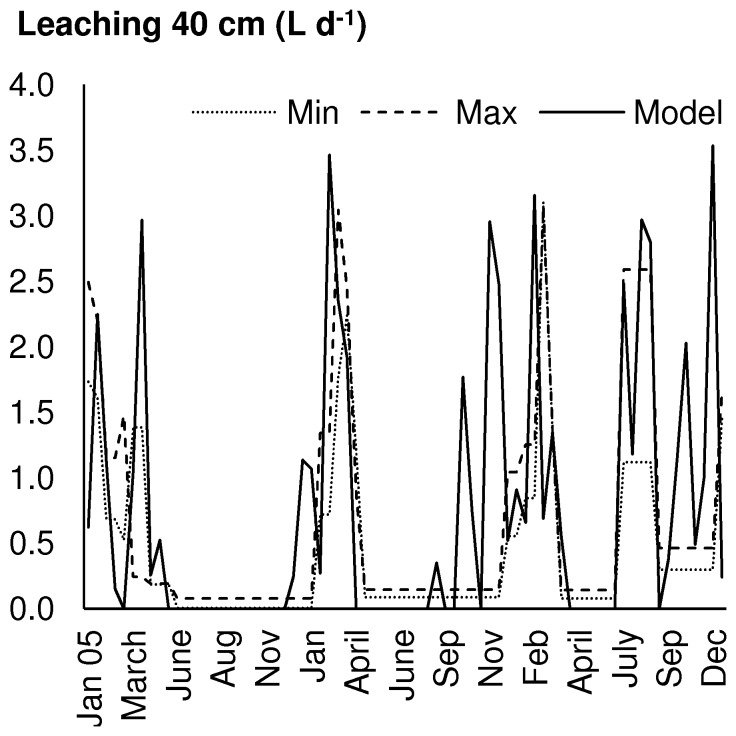
Leaching of water from soil layer 2. Model compared to measurement for CTR, MSW and GSW treatments. Model is average of all predictions, min and max is minimum and maximum lysimeter measurements, MSW(I) and CTR(II), respectively.

Calculated leaching of water from the second to the third soil layer was compared to water collected in situ at 40 cm depth with lysimeters, in 3 plots of the field (CTR, MSW and GSW) for the period from January 2005 to December 2007 [17] (Figure 5). Estimated leaching of 805, 815 and 819 L for the entire period for the GWS, MSW and CTR treatments are higher than the measured values (GWS: 474–535 L, MSW: 488–539 L, CTR: 648–741 L).

## Discussion

### Concept

The concept to couple the flux-based plant uptake model to a simulation model for water and solutes in discrete soil layers seems promising to us and allows the simultaneous simulation of water budget and plant uptake. The model can simulate various scenarios with different crops, soil and water conditions. However, the model has only one plant compartment (i.e. internal distribution is not accounted for) and the concept is limited to non-essential heavy metals, because the plant uptake of essential heavy metals from soil is regulated [4]. The full potential of the model concept could not be realized, because most soil properties (including concentrations) were determined only for the top soil. Thus, the simulations should rather be considered as illustrative.

### Accuracy of Predictions

The simulated concentrations of Cd and Pb in soil and plants can be compared to the measured ones. The predicted increasing trend for Cd in top soil is not seen in the measured data. The samples taken last (July 2007) show for all soil variants the lowest concentration (Figure 2). There is a significant correlation (p<0.05) between some of the measured concentrations from the five treatments (CTR-GWS, CTR-MSW, GWS-MSW and BIOW-MSW), indicating that the sampling or analysis method has some influence on the results. On the other hand, the measured concentrations are rather consistent (all median values range between 0.21 and 0.27 mg kg dw^−1^), which shows that the analytical method is precise. But not precise enough to show the small changes predicted by the model. For Pb, too, the measured soil concentrations from the last samples are comparatively low. All other measured data confirm the upward trend of top soil concentrations for amended soils. Measured Pb concentrations in control soil are the lowest, and have a constant trend. Predicted and measured concentrations are in this regard in good agreement.

The predicted concentrations of Cd in plants range between 0.025 and 0.085 mg kg dw^−1^, the measured ones in stems and leaves between 0.02 and 0.087 mg kg dw^−1^. For Pb, the predicted concentrations in plants are between 0.22 and 0.89 mg kg dw^−1^, those measured in stems and leaves between 0.2 and 1.08 mg kg dw^−1^. This is a rather good agreement, given the fact that the model is purely based on the calculation of passive transport with the water flux and deposition from air. In some cases, other factors than passive uptake with soil water may play a significant role, for instance the presence of competing ions like Ca^2+^ [18] and a high Cl^-^ content of soils [4]. However, these effects do not seem to be relevant in our study. Furthermore, the model allows an interpretation of the relevant processes: for Cd, uptake from soil is dominating, while for Pb, deposition from air is the most relevant uptake process for the first three years.

In some details, the model has limitations. Measured concentrations in grains, which are more relevant for human consumption than stems and leaves, are lower than the simulated concentrations in plants. One reason for this could be that the water within plants flows mainly to leaves, from where it evaporates, while grains receive less water (about 1–2% of the xylem flow) and additionally are supplied with phloem sap. Therefore, the relation between growth and water uptake, which was used to calculate the transpiration stream and passive uptake from soil, does not hold for grains. The model has only one plant compartment and is not intended to simulate the internal distribution of metals within the plant, such as decreased concentration with distance from the roots due to sorption [19]. For the transport of metals to the grain via phloem, enzymatic processes could be involved, because the phloem sieve tubes are living cells [20] and some studies have suggested that ions like Cu^2+^ and Zn^2+^ are competing with Cd in the transport to grains [21], [22]. In this study, measured concentrations of Cd and Pb in grains are mostly below the quantification limit and much lower than concentrations in stems and leaves. Mench et al. [21] also reported Cd content in wheat grains as being lower than that in the shoot, whereas Lavado et al. [15] found similar concentrations of Cd in both shoot and grain. It is uncertain, whether the modeling approach used here, i.e. physiologically based simulation of passive transport processes, can be modified so that it will successfully predict concentrations of non-essential metals in grains.

Another detail where the model does not meet the data is that measured concentrations of Cd are significantly (p<0.05) lower for wheat straw than for maize straw, about one third. The same is seen for Pb, but less pronounced the first years, when deposition from air plays a major role [23]. The predictions are opposite, because maize, as C4-plant [20] needs less water per produced biomass, and thus the passive transport of solutes into the plant is, relatively seen, less. The model offers no explanation for this deviation, but it is known that genetic factors influence uptake [14]. Also, Lavado et al. [15] measured the same trend as the model predictions with Cd concentrations being higher in wheat straw compared to maize straw. Measured concentrations in grains are in this study similar for maize and wheat, since most data are below or slightly above the quantification limit.

Concentrations of Cd and Pb in deeper soil layers and groundwater were in this study set equal to the concentration in top soil, since no measured data were available. However, this is probably not exact and we would expect lower concentrations in deeper layers. This would decrease the predicted concentrations in the soil solution of these layers, in leaching water and in plant tissues, since deep layers contribute to transpiration part of the year. E.g., by assuming the concentration in groundwater equal to half of the concentration in the lowest soil layer; Cd concentrations in maize are decreased by 16% for 1999.

The simulation of the water balance including leaching mimicked the timing and amount of soil water, as can be seen from the comparison of predicted and measured leaching of water (Figure 5). The model results were not fitted, and the measured results were only available after all simulations had been done. Two important simplifications were made with respect to the role of plants in the water balance. First, transpiration was calculated from measured plant growth data (Table 6), using a constant factor, the transpiration coefficient (Table 3). Second, and that is a novelty in the model approach, we skipped the calculation of root distribution, and assumed instead that roots grow to the soil layer where they find water [24]. Both assumptions avoid parameter-intensive calculations, and were a prerequisite for simulations with the available data set.

**Table 6:** Calculated total mass of harvested plant parts (from results in dry weight, please see Text S2, Table S4).

Date of harvest	Crops	Total plant mass (kg fw m^−2^)				
		GWS	BIOW	FYM	MSW	Control
20 Oct 1999	Maize	9.92	9.73	10.64	9.86	9.88
25 July 2000	Wheat	6.64	6.42	7.02	6.69	6.52
20 Oct 2001	Maize	9.26	9.18	9.18	9.55	8.91
20 July 2002	Wheat	5.22	5.75	5.68	6.05	5.05
15 Oct 2003	Maize	8.32	9.22	9.10	8.50	9.11
25 July 2004	Wheat	7.80	7.78	7.87	7.68	7.21
15 Oct 2005	Maize	8.75	8.63	8.71	8.15	7.81
19 July 2006	Wheat	7.00	6.70	7.26	6.89	6.42
19 July 2007	Barley	7.33	5.79	6.80	6.18	5.04

GWS: Co-compost of greenwaste and sewage sludge, BIOW: Biowaste compost, FYM: Farmyard manure and MSW: Municipal solid waste compost.

### Effect of Soil Amendments

The predicted increase in top soil concentrations of Cd and Pb was solely due to the application of Cd and Pb contained in the amendments (Table 2), whereas concentrations in control soils were predicted to decrease slightly or stay constant. For Cd, the BIOW, GWS and MSW soils in 2002 and all amended soils in 2007 had a statistically higher concentration of Cd than the control soil (*p*<0.05). For Pb, the BIOW, GWS and FYM soils in 2006 and the BIOW, FYM and MSW soils in 2007 had a statistically higher concentration of Pb than the control soil (*p*<0.05). But despite this increase for soil, both measured and simulated concentrations in harvest products from the amended plots did not increase. The opposite was observed: simulated concentrations of Cd in plants increased for the control soil (and GWS amendment), but it decreased for the BIOW, MSW and the FYM amendment. Measured and simulated concentrations of Pb fell in harvest products from all plots (Figure 3). The reason is that deposition from air, which was responsible for higher concentrations in plants the first years decreased dramatically (concentrations of Pb in air were factor 21 higher in 1998 than in 2007). For Cd, deposition from air played only a minor role, and predicted concentrations in harvest products fell due to increasing *K_d_*. All amendments increased the organic carbon content of the soil, while it fell slightly in the control soil (Table S1). Three of the amendments (FYM, BIOW, MSW) furthermore increased pH. Consequently, the calculated *K*_d_ of Cd and Pb increased in these three soils. For the GWS soil, only the *K*_d_ of Cd at the end was the same as initial (but with falling trend in between), the *K*_d_ of Pb fell. The calculated *K*_d_ of both metals decreased in control soil over the ten-years period (Table S1), leading to a predicted increase in plant uptake. This means, within the considered period, the application of FYM, BIOW and MSW amendments led to a reduction of the simulated heavy metal content in harvested crops. However, the organic carbon may be degraded again. In control soils, the average organic carbon fell from 1.072 g g^−1^ in 1998 to 1.045 g g^−1^ in 2007.

### Comparison to Other Findings

The reduction of bioavailability of heavy metals by soil amendments was also mentioned in the review of Smith [2]. Accordingly, compost typically increases pH. A study comparing MSW amended soil to soil receiving mineral salts found a slight increase of soil concentrations but reduced transfer of Pb and Cd to field grown fodder crops from the MSW amended soil after 4 years of application, compared to mineral salt fertilized soils [25]. Similar, Gondek et al. [26] found no difference in aboveground concentrations of Pb in maize for maize grown in sewage sludge amended soils, compared to maize grown in soils fertilized with minerals only.

### Comparison to Other Model Approaches

A variety of approaches is used to predict the uptake of heavy metals from soil into crops [4]. Commonly used are empirical bioconcentration factors (BCFs). These BCFs often have the form of a regression between soil concentration, soil properties and concentration in plants and are easy to apply. The disadvantage of such regressions is that they are typically limited to their regression range, and often only hold for a certain type of plant species, and within a limited range of soil properties. In a recent study, we could not confirm that multi-parameter regressions are superior to simple empirical, crop-specific transfer factors [5]. The model applied in the present study belongs to the so-called physiological models [4]. Their advantage is that conditions at site (such as plant growth and water budget) can be considered, and may explain uptake differences between the years. Peijnenburg et al. [27] used soil pore water concentrations and the water use efficiency (which is the inverse of the transpiration coefficient used in our study) multiplied with the weight change of plants to predict successfully the uptake of Cd and Zn into lettuce. Also, Ingwersen and Streck [28] estimated the concentration of Cd in wheat, sugar beet and potato using transpiration, concentration in soil solution and a plant specific empirical uptake efficiency parameter. For wheat, passive uptake was assumed and the uptake efficiency set to one. These approaches are thus very similar to the one used here. A difference is that we additionally considered deposition from air, but this process was only relevant for Pb. A more complex approach is the *Barber-Cushman* model which simulates advection and diffusion into roots using root geometry and soil properties by solving the underlying partial differential equation [29]–[31]. The approach may be useful to explain uptake processes of nutrients and heavy metals, but it is troublesome to derive the required input parameters on a field scale [4].

The coupling of physiological plant uptake models with water and solute transport models for soil is rare. Bauer-Gottwein et al. [9] combined a groundwater transport model with a physiological model for salt uptake and simulated the formation of salt islands in the Okawango delta. No publication about an approach to couple heavy metal transport in soil and groundwater to physiological plant uptake models is known to us. Therefore, our approach is probably unique. A common problem, namely the description of root distribution, root growth and root water uptake, was solved by the following assumption: Roots grow to where the water is; roots take up water from the highest soil layer where water is available; if this layer is depleted, roots continue to take up water from the next (deeper) layer. This description may be oversimplified in some cases, e.g., when the water content of the soil with depth changes rapidly. This may, for example, happen when precipitation events with high intensity appear after longer periods of drought. On the other hand, this algorithm allows an easy and efficient description of otherwise quite complex and largely unknown processes.

### Conclusions

The long-term simultaneous simulation of the water budget of soil, the uptake into plants and the leaching to groundwater of two heavy metals on field scale succeeded by coupling a physiological plant uptake model to a buckets soil model. Concentration in soil of Cd and Pb, plant uptake, leaching, and deposition from air were simulated for a ten-years field experiment where biowaste compost, municipal solid waste compost, co-compost of green waste and sewage sludge and farm yard manure were applied to soil. In top soils from the control plot, calculated concentrations of Cd were slowly declining (2.6% in 10 years), mainly due to leaching. The calculated concentration of Pb in the control top soil was practically constant (+0.2%).

When soils were amended, calculated Cd and Pb concentrations in top soil were in all cases increasing, about 10% for Cd, and between 6% and 18% for Pb. Most organic soil amendments led to a reduction in the simulated plant uptake, because soil pH and organic carbon and thus the calculated *K*_d_ was increasing. Deposition from air was the dominating process for Pb before 2001, but hereafter was less relevant, due to steeply declining concentrations of Pb in air [16]. The comparison between simulated and measured concentrations in soils and plants showed overall good agreement, but also deviations in details. The uptake into plants using water flux and heavy metal concentration in soil pore water yielded concentrations which are comparable to those measured in leaves and stems, but the approach does not seem applicable for concentrations in grains.

The model can predict other scenarios and future trends for plant uptake and leaching of Cd and Pb. Also, future work should focus on variation of concentration in soil with depth and the possibility of adding an extra plant compartment to the plant model, so that the concentration in grains can be predicted.

## Materials and Methods

### Field Study

**Figure 6:**
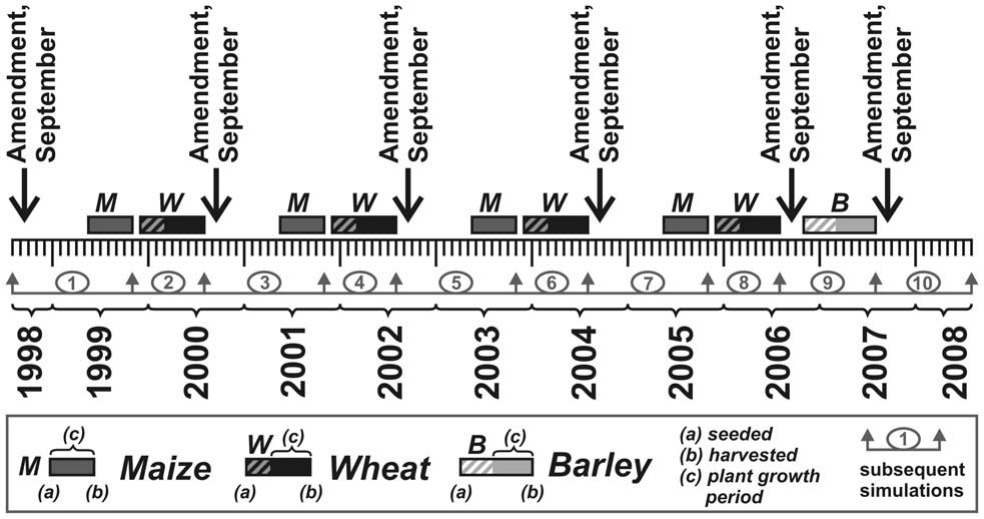
Overview of the field and simulation study. For wheat and barley the starting point of growth takes place after seeding.

The “QualiAgro” long term field experiment has been initiated in 1998 by INRA de Grignon and Veolia Environnement R&I in order to study the benefits and environmental impacts of repeated urban compost applications on soil, water and plant qualities. The field is located at Feucherolles, Ile de France, 35 km west of Paris and is equipped with a meteorological station nearby that records climatic parameters [3]. Mean annual temperature is 11°C. Mean annual rainfall amounted to 582 mm yr^−1^ (average data between 1989 and 2009 measured in the nearby weather station). The soil is a silt loam Luvisol and contains on average 15% clay, 78% silt and 7% sand in the ploughed layer. Amendments included three urban composts (bio waste compost, BIOW; municipal solid waste compost, MSW; co-compost of green waste and sewage sludge, GWS) as well as farm yard manure (FYM). Applications were compared to controls without amendment (CTR). The experimental field was divided into 20 plots of 450 m^2^ with 4 replicates for each treatment (the four amendments and the control). Amendments were applied and incorporated to soil in September 1998, 2000, 2002, 2004, 2006 and 2007 after wheat harvest. Composts and manure were applied at doses equivalent to 4 t carbon ha^−1^, corresponding to 15 to 20 t dry weight (DW) ha^−1^ depending on the organic products (Table 2). In May the following year (1999, 2001, 2003 and 2005) maize was seeded and it was harvested in October, giving it a growth period of 5.5 months (169 days). After harvest of maize, wheat was seeded in November (1999, 2001, 2003 and 2005) and harvested in July of the following year, assuming starting point of growth in March and thereby a growth period of 5 month (150 days). Barley was only seeded in October 2006, 1 month after application of amendment and harvested in July 2007. It was seeded in replacement of maize, because of a pest (*Diabrotica virgifera*) alert. Additional mineral N fertilizer was added in each treatment to reach optimum crop yield. Figure 6 gives an overview on amendment application and succession of crop cultivation. Plants (harvested grains and plant residues) were analyzed for metals and other characteristics. Wheat residues were always exported and maize residues incorporated into soils after harvest. Sampling of soil plough layers and organic amendments was done prior to each amendment application, in early September or late August. For each plot a representative soil sample was obtained from 10 sampling points. Amendments were sampled in triplicates. All samples were conditioned and analyzed for metal content and other parameters according to normalized methods (see Chemical analyses section). The median of the replicates (three for amendment and 4 for soil and plant samples) is reported here. Three plots of the field, corresponding to the 3 treatments CTR, MSW and GWS, were equipped by mid 2004 with lysimeters at a depth of 40 cm in order to collect soil water during the drainage periods [17].

All necessary permits were obtained for the described field studies. An agreement was made between the land owner, Mr Bignon, and INRA.

### Chemical Analyses

All analyses of soils and amendments were performed at the INRA Laboratoire d’Analyses des Sols (Arras, France). All analyses of plants were performed at the USRAVE laboratory (INRA Bordeaux, France). Both laboratories are accredited according to NF ISO/CEI 17025 for the soil and plant analyses reported here. Concerning the analysis of amendments, INRA Arras applies the same quality controls and the same validation methods (norm NF V3-110 and T90-210) as for soil analysis.

Soil samples were dried at 40°C and passed through a 2 mm sieve. Representative aliquots were ground and sieved at 250 µm before C and total metal analyses. Organic C was determined by catalysed combustion-oxidation (norm ISO 10694). Metal analyses were performed by ICP-MS after heating at 450°C and complete digestion in HF-HClO_4_ (norm NF X 31–147). pH was measured on a 10 g aliquot of the sample <2 mm dispersed in pure water (soil:water 1∶5; norm ISO 10390). Exchangeable metals were determined from extraction in 0.01 M CaCl_2_ solution (ratio 1∶10, shaking 2 h, centrifugation and filtration; NL norm NEN 5704).

Organic amendment samples were freeze-dried, ground and sieved at 5 mm. Total metals and C were analysed on aliquots like for soils. The carbonate content was also analysed and, when significant, inorganic C was subtracted from total C to get the organic C. Total metal contents were obtained by the same digestion method as for soil followed by ICP-AES (NF ISO 2203).

Plant samples were ground and homogenized with a rotary homogenizer. 1 gram of dry plant powder was weighed in a silicon capsule and incinerated in a muffle furnace at 480°C for 5 h. Afterwards, it was digested with concentrated nitric acid in several steps. The remaining powder collected on ash-free paper was incinerated at 550°C for 2 h. Ashes were dissolved in a Teflon capsule by 5 mL of concentrated hydrofluoric acid, evaporated and dissolved again in two steps with concentrated nitric acid. All obtained solutions were collected in a volumetric flask and completed to 100 mL with distilled water. An ICP-AES Iris Intrepid (Thermo Fischer Scientific Inc., Waltham, USA) and an ICP-AES Liberty Serie 2 (Varian, Mulgrave, Australia) equipped with an ultrasonic nebulizer U-5000AT+ (CETAC, Omaha, Nebraska, USA) was used for Cd and Pb analysis. Spectrometer operating conditions are fully described elsewhere [32], [33]. Analysis quality was controlled using an in-house laboratory reference sample V463 (entire maize plant) and blanks which have undergone the entire analysis process. Concentration values measured in blanks were subtracted from concentration values measured in the samples.

All chemical contents were expressed per dry weight (dw) 105°C according to the norms NF ISO 11465 and NF U44-171 for soils and amendments, respectively. The weighing and humidity correction for plants were done using a meteorologically controlled scale (Mettler Toledo S.A., Viroflay, France) and a meteorologically controlled drying cupboard at the temperature of 103±5°C.

From 1999 to 2005, the quantification limits (QLs) in plants were ≤0.03 mg kg dw^−1^ for Cd and ≤0.2 mg kg dw^−1^ for Pb. In 2006 and 2007, probably due to a temporary change of method, QLs were much higher, i.e. up to 0.3 and 1 mg kg dw^−1^ for Cd and Pb, respectively. In 2008 and 2009 (data not shown), QLs were again down to previous levels (0.03 mg kg dw^−1^ for Cd and 0.2 mg kg dw^−1^ for Pb). Only the QLs from 1999–2005 and 2008–2009 were applied in this manuscript. QLs for the total metal contents of soil are 0.02 mg Cd and 0.2 mg Pb per kg dw (down to 0.1 mg Pb per kg dw after 2006). For organic amendments QLs are higher, 0.5 mg Cd and 2 mg Pb per kg dw. For aqueous samples, QLs are 0.05 µg L^−1^ of Cd and 0.2 µg L^−1^ of Pb.

### Modeling Approach

**Figure 7:**
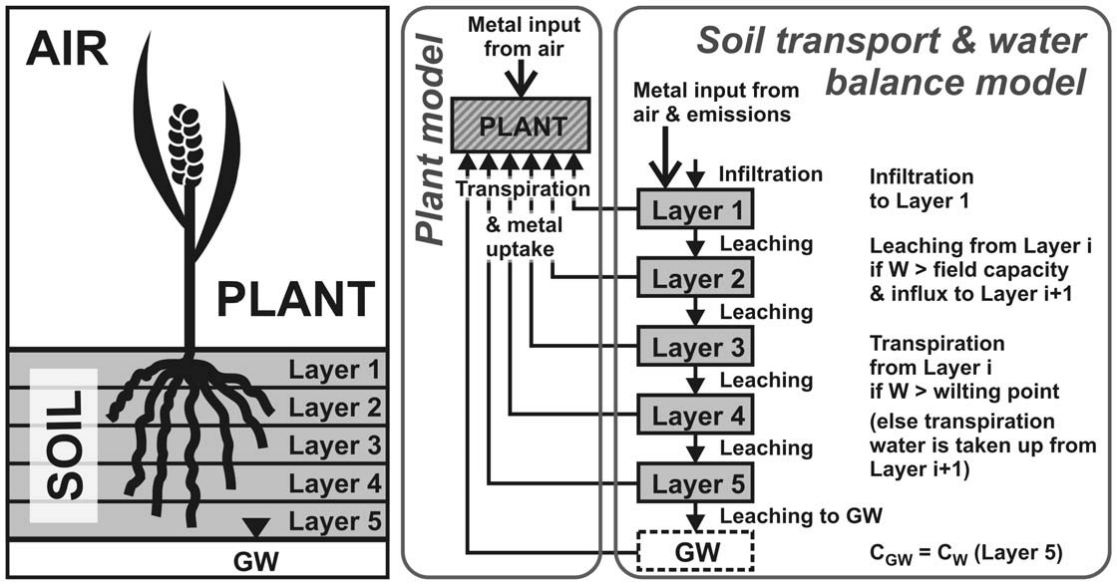
Processes and compartments in the coupled soil solute transport, water balance and plant uptake model. W: water content, GW: groundwater, C_GW_: groundwater concentration, C_W_: soil pore water concentration.

Modeling of metal transport in the soil-air-plant system was done by coupling a model for water and solute transport in soil including a discrete cascade approach for the water balance (tipping buckets model) [12], [34] to a dynamic plant uptake model similar to the multi-cascade approach [10], [11] (Figure 7). The tipping buckets soil water and substance transport model was chosen because its step-wise and periodic simulation mode makes it easily compatible to the step-wise solution method of the analytical multi-cascade plant model. A second reason was that the time period for simulation was ten years, and the buckets approach needs only a reasonable number of input data. In each time period, the water and substance balance in the five soil layers is solved iteratively considering precipitation, infiltration, leaching and transpiration (i.e., water uptake from soil by growing plants). Uptake of heavy metals into plants is with the water taken up by the roots at various depths. The coupled soil water and solute transport and plant uptake model was realized as Microsoft Excel™ spreadsheet.

### Tipping Buckets Model for Transport of Water in Soil

The discrete tipping buckets water balance model [12], [34] considers *m* soil layers located above the groundwater table, for which the water balance is calculated. Five soil layers (*m* = 5, Table 4) were specified in the applied model. The soil layers are considered to be a series of “tipping buckets”, which have an upper and lower limit for water storage capacity: the water content at the upper limit is the field capacity, *FC* (L), and that at the lower limit is the permanent wilting point, *PWP* (L). Flow is discontinuous, i.e. the soil layers are considered as buckets that can be filled up to field capacity, after which they tip, and by putting the soil layers in series, tipping buckets arise that transport water and solutes. The model considers downwards (leaching) as well as upwards (transpiration and evaporation) movement of water and solutes. Transpiration, i.e. water extraction by plants, is calculated from plant growth (see later section). It is assumed that plant roots always extract water from the highest possible soil layer, and until the *PWP* is reached [8]. Capillary rise from the groundwater table to the plant roots was not included in the model, except as part of the transpiration in the growing season of the plants. Also, groundwater elevation due to leaching was neglected. Precipitation, evaporation and transpiration were considered and each calculation was done in eight steps as detailed in the following. All calculations were done for an area of 1 m^2^.

#### Step 1

Initial (absolute) water content in the top soil layer (soil layer 1), *W_Ini1_* (L), is obtained from initial volumetric water content, *θ_W,Ini1_* (L L^−1^), and the volume of soil layer 1, *V_S1_* (L), as
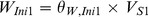
(1)


#### Step 2

Infiltration, *Inf* (L d^−1^), is calculated from precipitation, *P* (L d^−1^), and evaporation, *E* (L d^−1^), (soil layer 1):
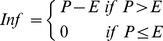
(2)


#### Step 3

After infiltration, a new water content, *W_Inf1_* (L), is established in soil layer 1:
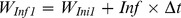
(3)where Δ*t* (d) is the length of the time period.

#### Step 4

Leaching from soil layer 1, *Leach_1_* (L), occurs if the water content is now above field capacity *FC* (L):
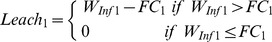
(4)


#### Step 5

After leaching, the water content of soil layer 1 changes to *W_Leach1_* (L):
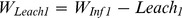
(5)


#### Step 6

Transpiration, i.e. water flux to plants from soil layer 1, *q_1_* (L), takes place if the water content is now above the permanent wilting point *PWP_1_* (L):
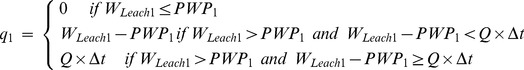
(6)where *Q* (L d^−1^) is the total transpiration of the plant in this period (see later section).

#### Step 7

After transpiration, again a new water content, *W_q1_* (L), is established in soil layer 1:
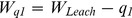
(7)


#### Step 8

Finally, remaining transpiration *q_Total-1_* (L), i.e. transpiration water that needs to be taken from deeper soil layers, is obtained by:
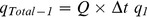
(8)


For the next soil layers (soil layer *i*, with *i*>1), steps 3 to 8 are repeated. However, Step 3 (Eq. 3) is the new water content of layer *i* due to leaching from above:
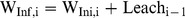
(9)


Step 6 (Eq. 6) changes to
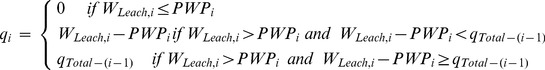
(10)and Step 8 (Eq. 8) changes to



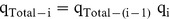
(11)The water balance was established iteratively for all soil layers *i* in each time period *p*. The calculated water content after transpiration from one time period, *W_q,i,p_*, was entered as initial water content for the following time period, *W_Ini,i,p+1_*.

If the plant does not find sufficient water in the five soil layers (i.e., Q>∑q_i_), it is assumed that the remaining water required for transpiration is drawn from groundwater. This does not affect water or substance content of the five soil layers. In the present model formulation we assume that the groundwater has the same substance concentration as the water in the lowest soil layer.

### Solute Transport in Soil

Solutes passively follow the water movement. The change of solute concentration in soil is given by input from air and pulse emissions (amendment application) to soil layer 1 minus loss of solute by leaching and plant uptake via transpiration. As heavy metals are considered in this study, loss by degradation and by volatilization from the top layer is not of relevance. In discrete form, the concentration in soil layer 1, C^*^_S,1_ (mg L^−1^) (referred to the volume of bulk soil, *V*_S_), at time *t* is:
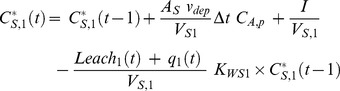
(12)where C^*^_S,1_(*t*−1) is metal concentration in soil layer 1 at time *t*−1 (preceding time period), *A_S_* (1 m^2^) is the surface area of the soil, *v_dep_* (m d^−1^) is the deposition velocity of particles, *C_A,p_* (mg m^−3^) is the total concentration (usually at particles) in air and *I* (mg) is the pulse input of metal (from amendment application). The water to dry soil partition coefficient *K_WS1_* (−) in soil layer 1 was calculated as
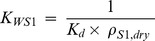
(13)where Kd (L kg dw−1) is the dry soil to water partition coefficient and ρS,dry (kg dw L−1) is the density of dry soil.

The change of metal concentration in the second and following soil layers (index *i*, with *i*>1) is given by influx of solute from the upper soil layer via leachate minus loss by leaching to deeper soil layers and transpiration. Soil concentration C^*^_S,i_ at time *t* is accordingly:
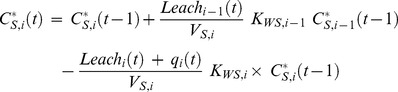
(14)


The volume-based concentrations in bulk soil, C^*^_S_ (mg L^−1^), can be converted to soil dry weight, *C_S_* (mg kg dw^−1^), by dividing by the dry soil density, *ρ_S,dry_* (kg dw L^−1^). For solutes, the Courant criterion [12] needs to be fulfilled, which says that in one step not more compound can flow out of a layer than is in it. This limits thickness of the layers and length of time steps.

### Plant Growth and Transpiration

Transpiration was coupled to plant growth and implemented in the model according to Rein et al. [11]. Logistic plant growth was assumed (following e.g. Richards [35]), where the change of plant mass *M* (kg fw) with time *t* (d) can be expressed as
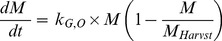
(15)where *k_G,O_* (d^−1^) is the overall first-order growth rate constant and *M_Harvest_* (kg fw) is harvested (assumed maximum) plant mass. With initial plant mass *M_Initial_* (kg fw) (plant mass at time *t* = 0), the analytical solution is:



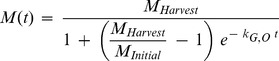
(16)Transpiration, *Q* (L d^−1^), is coupled to plant mass growth via the transpiration coefficient, *T_C_* (L kg fw^−1^) [8]. In discrete form, transpiration *Q* at time *t* induced by changing (growing) plant mass is accordingly given by
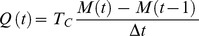
(17)where *M*(*t*) and *M*(*t*−1) are plant mass (kg) at time *t* and *t*−1 (preceding time period) and Δ*t* (d) is the length of the time period. First-order growth rate constants, *k_G_*(*t*) (d^−1^), specific to each time period were obtained by



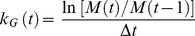
(18)These were used for step-wise (i.e. time-period-wise) approximation of logistic growth and applied as first-order loss rate constants for growth dilution (see Eq. 19; please refer to Rein et al. [11] for more details). The application of these formulae requires only four input data (*M*_Initial_, *M*_Harvest_, *k*_G_ and *T*_C_) for the whole simulation, instead of plant mass and transpiration data for each period.

### Plant Uptake Model for Non-essential Metals

Non-essential heavy metals show plant uptake linearly related to their concentration in soil solution [4]. We assumed passive uptake of heavy metals with soil water into the plant. The transpiration of the plant depends on its transpiration coefficient and growth (see above). The model contains only one plant compartment and the change of concentration in the plant compartment was calculated from input via wet and dry particle deposition plus input via uptake from soil minus loss by growth dilution:
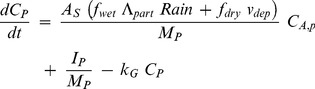
(19)where *C_P_* (mg kg fw^−1^) is the concentration of metal in plant tissue, *Λ_part_* (m^3^ air m^−3^ rain) is the rainfall scavenging ratio for particles, *Rain* (m d^−1^) is precipitation, *M_P_* (kg fw) is plant mass, *I_P_* (mg d^−1^) is the uptake of metal from soil and *k_G_* (d^−1^) is the first-order growth rate constant of plants (for consideration of growth dilution). This equation can be used to predict the overall concentration in plants.

The fractions of metal in rainfall and at particles that are intercepted by and transferred to the plant, *f_wet_* and *f_dry_* (−), were calculated from plant mass and absorption coefficients, *µ_wet_* and *µ_dry_* (m^2^ kg dw^−1^) [36]:
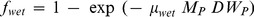
(20a)

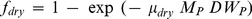
(20b)where *DW_P_* (kg dw kg fw^−1^) is the dry matter content of the plant, which is equal to one minus the water content of the plant (1−*W_P_*) and *M_P_* is plant mass in units of kg fw m^−2^ (for an area of 1 m^2^). The uptake of metal from soil into plant, *I_P_* (mg d^−1^), in each period was calculated as the sum of the uptake from all *m* soil layers and from groundwater via transpiration, *q* (see above):
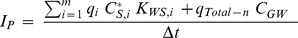
(21)where CGW (mg L−1) is groundwater concentration, which is assumed equal to pore water concentration in the deepest soil layer (CGW = C*S,n x KWS,n), and qTotal-n (L) is the remaining transpiration of the plant that cannot be satisfied from soil water. Attachment of soil particles was considered subsequently as an additional process, assuming that a fraction of soil particles (default 0.1% for cereals) is attached to plant surfaces [37]:



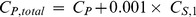
(22)For the dynamic calculation of the concentration in plant, the principle of superposition was applied, i.e. the simulation was divided into *n* periods during which all parameters are kept constant (each period was then further subdivided into 30 time intervals, at which intermediate results were calculated). This procedure allowed the application of analytical solutions of the differential equation (Eq. 19) for each period, i.e. the result from one period is entered as initial value for the following period. This also allowed varying all rates and constants from period to period, and thus, to model time-varying contaminant input as well as to approach non-linear input (such as logistic growth of plants, or changing weather conditions).

### Simulation Study and Model Parameterization

The total simulation was ten years (August 1998 to July 2008) and was subdivided into ten consecutive simulation events, each ending with harvest (Figure 6). The simulation events were further subdivided into periods of two weeks (first and second half of each month). Five simulations were carried out, one for the control plot (the only source of pollutants for crops can be the background level or the aerial deposition) and one for each type of amendment (bio waste compost, BIOW; municipal solid waste compost, MSW; co-compost of green waste and sewage sludge, GWS and farm yard manure, FYM).

#### Soil data

Density, porosity, thickness of soil layers, field capacity and permanent wilting point of the soils were considered equal for all soils (Table 4). The simulation in August 1998 started with “empty” soil, i.e. the water content of all soil layers was set to the permanent wilting point, corresponding to the typical situation towards the end of the growing season. For all other years, the initial water content of the soil layers was the calculated final water content of the year before.

Total metal contents of soils measured in 1998, before the first amendments, slightly differed among the five treatments; the median values from the 4 field replicates were input for the simulation (Table S1). For the following years, the calculated concentrations of the year before served as starting concentration. Organic carbon content and pH varied slightly with plot and year (Table S1). The soil to water distribution coefficients, *K_d_* (L kg dw^−1^), for Cd and Pb, was estimated by the following regressions [7]:
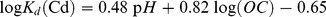
(23)

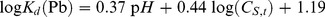
(24)where p*H* is the p*H* of soil water, *OC* (% (dw dw^−1^)) is the percentage of organic carbon in soil and *C_S,t_* (mg kg dw^−1^) is the measured total concentration of metal in soil (Table S1). Measured data for the top soil were applied to estimate soil concentrations and *K_d_* for all five soil layers (Table 4).

#### Water balance

Recorded daily precipitation rates representative for the QualiAgro site were averaged to give one precipitation estimate per half month (Table S2). Evaporation from soil, *E* (L m^−2^ d^−1^), was estimated from reference evapotranspiration, *ET_0_* (L m^−2^ d^−1^), as
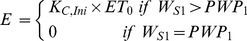
(25)where *K_C,Ini_* (−) is the crop coefficient from the initial growth stage of the crop (a value of 0.3 used as best estimate for cereal crops [38]). Reference evapotranspiration, averaged for 15 days, was calculated using the Penman-Monteith equation [39] (Method see Text S1, results Table S3). Transpiration by plants was calculated as described above. Surface run-off of water was neglected.

#### Plants

Crop-specific parameters are transpiration coefficient, growth rates and initial and final plant mass (Table 3, Text S2, Table S4). The harvested amounts of grains and residues (consisting of leaves and stems) were measured in the field experiment on a dry weight basis (Table 6).

#### Air

Concentrations of Cd (0.68 ng m^−3^ in 1999 to 0.28 ng m^−3^ in 2008) and Pb (0.21 µg m^−3^ in 1998 to 0.01 µg m^−3^ in 2007) measured in air in Paris were taken as input parameters [16]. For the calculation of particle deposition, a rainfall scavenging ratio was applied (*Λ_part_* in Eq. 1); literature values range from 1000 to 200 000 [12], and a value of 20 000 m^3^ m^−3^ was chosen. The default particle deposition velocity (*v_dep_*) is 0.001 m s^−1^ (fine particles [12]).

#### Input via amendment

The input (mg m^−2^) of Cd and Pb to soil via amendment is simulated as pulse input by the model. Data in Table 2 were derived from measured amendment concentrations by multiplying with applied amendment mass.

## Supporting Information

Table S1 **Estimation of soil-water partition coefficient** ***K_d_*** **.** Measured concentration of Cd and Pb in soils, organic carbon content and pH of soils together with estimated *K_d_*’s from Sauvé et al’s equations.
(DOCX)

Table S2 **Precipitation.** Precipitation rates (m d^−1^), average half monthly values from August 1998 to December 2002 (1998 to August 2002: records from Parc Meteo Grignon, Grignon, France, about 5 km southwest of the test site; November 2002 to 2008: records from Feucherolles, France, adjacent to the test site).
(DOCX)

Table S3 **Evaporation.** Calculated evaporation rates (0.3×*ET_0_*) (L m^−2^ d^−1^), average half monthly values from August 1998 to July 2008.
(DOCX)

Table S4 **Plant mass.** Estimated grain, leaf, stem and root mass (mg kg fw m^−2^).
(DOCX)

Text S1 **Evaporation.** Method for calculating reference evapotranspiration.
(DOCX)

Text S2 **Plant mass.** Method for estimating plant mass.
(DOCX)
