# Supplementary material for: Simultaneous Simulations of Uptake in Plants and Leaching to Groundwater of Cadmium and Lead for Arable Land Amended with Compost or Farmyard Manure
Source: PLoS One. 2012 Oct 4;7(10):e47002. doi: 10.1371/journal.pone.0047002 (PMC3464289; doi:10.1371/journal.pone.0047002)
Supplement: Table S1 — Estimation of soil-water partition coefficient Kd . Measured concentration of Cd and Pb in soils, organic carbon content and pH of soils together with estimated Kd’s from Sauvé et al’s equations. (DOCX) [file pone.0047002.s001.docx]

**Estimation of soil-water partition coefficient *K_d_***

Soil measurements were performed in 1998, 2000, 2002, 2004, 2006 and 2007 in early September (except for 2004 in late August) before application of amendment. Estimated Kd’s from 1998 were used for the two subsequent simulation sequences from August 1998 to July 2000, estimated Kd’s from 2000 were used for the two subsequent simulation sequences from August 2000 to July 2002, and so forth.

**Table S1.** Measured concentration of Cd and Pb in soils, organic carbon content and pH of soils together with estimated *K_d_*’s from Sauvé et al’s equations.

| **Soil** | **Year** | ***C_Soil_* (Cd)**  **(mg kg dw^-1^)** | ***C_Soil_* (Pb)**  **(mg kg dw^-1^)** | ***OC***  **(% (dw dw^-1^))** | **p*H***  **(-)** | ***K_d_* (Cd)**  **(L kg dw^-1^)** | ***K_d_* (Pb)**  **(L kg dw^-1^)** |
| --- | --- | --- | --- | --- | --- | --- | --- |
| Control | 1998 | 0.24 | 24 | 1.1 | 7.1 | 609 | 26 092 |
|  | 2000 | 0.25 | 24 | 1.1 | 7.1 | 573 | 25 256 |
|  | 2002 | 0.23 | 25 | 1.1 | 7.1 | 542 | 25 277 |
|  | 2004 | 0.23 | 26 | 1.0 | 6.9 | 463 | 22 957 |
|  | 2006 | 0.22 | 24 | 1.0 | 6.9 | 424 | 21 952 |
|  | 2007 | 0.21 | 22 | 1.0 | 6.8 | 423 | 19 575 |
| GWS | 1998 | 0.23 | 33* | 1.0 | 7.1 | 588 | 28 797 |
|  | 2000 | 0.27 | 25 | 1.1 | 6.9 | 502 | 22 777 |
|  | 2002 | 0.24 | 23 | 1.1 | 7.0 | 514 | 23 006 |
|  | 2004 | 0.24 | 27 | 1.2 | 6.8 | 489 | 22 843 |
|  | 2006 | 0.23 | 27 | 1.2 | 6.8 | 457 | 21 119 |
|  | 2007 | 0.22 | 24 | 1.4 | 6.9 | 588 | 21 242 |
| FYM | 1998 | 0.24 | 25 | 1.1 | 7.0 | 538 | 24 695 |
|  | 2000 | 0.24 | 26 | 1.1 | 7.1 | 624 | 27 402 |
|  | 2002 | 0.25 | 26 | 1.2 | 7.2 | 710 | 27 742 |
|  | 2004 | 0.24 | 28 | 1.2 | 7.2 | 744 | 31 049 |
|  | 2006 | 0.23 | 28 | 1.2 | 7.2 | 779 | 30 416 |
|  | 2007 | 0.23 | 25 | 1.3 | 7.2 | 858 | 30 551 |
| BIOW | 1998 | 0.23 | 25 | 1.1 | 7.3 | 785 | 32 038 |
|  | 2000 | 0.24 | 25 | 1.1 | 7.3 | 720 | 30 551 |
|  | 2002 | 0.24 | 25 | 1.2 | 7.4 | 871 | 33 417 |
|  | 2004 | 0.24 | 27 | 1.2 | 7.4 | 904 | 35 528 |
|  | 2006 | 0.23 | 27 | 1.2 | 7.6 | 1146 | 41 715 |
|  | 2007 | 0.23 | 26 | 1.4 | 7.7 | 1437 | 44 652 |
| MSW | 1998 | 0.23 | 24 | 1.1 | 7.0 | 507 | 23 286 |
|  | 2000 | 0.25 | 23 | 1.1 | 7.3 | 727 | 29 821 |
|  | 2002 | 0.24 | 26 | 1.1 | 7.4 | 907 | 36 440 |
|  | 2004 | 0.24 | 29 | 1.1 | 7.4 | 843 | 36 053 |
|  | 2006 | 0.22 | 27 | 1.2 | 7.4 | 1028 | 38 034 |
|  | 2007 | 0.22 | 27 | 1.2 | 7.4 | 965 | 37 402 |

*C_Soil_*, *OC* and p*H*: Median values from 4 measurement replicates. *K_d_*: Median values from 4 estimates.*Value from the following measurement (25) were input to the model.
