# Supplementary material for: Simultaneous Simulations of Uptake in Plants and Leaching to Groundwater of Cadmium and Lead for Arable Land Amended with Compost or Farmyard Manure
Source: PLoS One. 2012 Oct 4;7(10):e47002. doi: 10.1371/journal.pone.0047002 (PMC3464289; doi:10.1371/journal.pone.0047002)
Supplement: Table S2 — Precipitation. Precipitation rates (m d−1), average half monthly values from August 1998 to December 2002 (1998 to August 2002: records from Parc Meteo Grignon, Grignon, France, about 5 km southwest of the test site; November 2002 to 2008: records from Feucherolles, France, adjacent to the test site). (DOCX) [file pone.0047002.s002.docx]

**Precipitation**

**Table S2**: Precipitation rates (m d^-1^), average half monthly values from August 1998 to December 2002 (1998 to August 2002: records from Parc Meteo Grignon, Grignon, France, about 5 km southwest of the test site; November 2002 to 2008: records from Feucherolles, France, adjacent to the test site).

| **Year** | **Month** | **1^st^ half** | **2^nd^ half** | **Year** | **Month** | **1^st^ half** | **2^nd^ half** |
| --- | --- | --- | --- | --- | --- | --- | --- |
| 1998 | August | 0.003 | 0.001 | 2001 | January | 0.002 | 0.002 |
|  | September | 0.004 | 0.001 |  | February | 0.002 | 0.0003 |
|  | October | 0.002 | 0.006 |  | March | 0.003 | 0.005 |
|  | November | 0.001 | 0.001 |  | April | 0.003 | 0.002 |
|  | December | 0.001 | 0.002 |  | May | 0.001 | 0.0001 |
| 1999 | January | 0.001 | 0.002 |  | June | 0.001 | 0.0004 |
|  | February | 0.001 | 0.003 |  | July | 0.008 | 0.001 |
|  | March | 0.001 | 0.001 |  | August | 0.004 | 0.0003 |
|  | April | 0.003 | 0.001 |  | September | 0.002 | 0.004 |
|  | May | 0.001 | 0.003 |  | October | 0.001 | 0.003 |
|  | June | 0.002 | 0.0002 |  | November | 0.002 | 0.002 |
|  | July | 0.001 | 0.0003 |  | December | 0.001 | 0.002 |
|  | August | 0.006 | 0.0003 | 2002 | January | 0.001 | 0.001 |
|  | September | 0.003 | 0.005 |  | February | 0.004 | 0.002 |
|  | October | 0.001 | 0.002 |  | March | 0.002 | 0.0052 |
|  | November | 0.002 | 0.001 |  | April | 0.00004 | 0.001 |
|  | December | 0.003 | 0.006 |  | May | 0.002 | 0.002 |
| 2000 | January | 0.001 | 0.001 |  | June | 0.002 | 0.001 |
|  | February | 0.002 | 0.002 |  | July | 0.004 | 0.001 |
|  | March | 0.001 | 0.002 |  | August | 0.002 | 0.002 |
|  | April | 0.003 | 0.003 |  | September* | 0.002 | 0.004 |
|  | May | 0.004 | 0.002 |  | October* | 0.001 | 0.003 |
|  | June | 0.003 | 0.00004 |  | November | 0.006 | 0.002 |
|  | July | 0.006 | 0.003 |  | December | 0.001 | 0.003 |
|  | August | 0.001 | 0.003 |  | | | |
|  | September | 0.002 | 0.001 |  |  |  |  |
|  | October | 0.004 | 0.004 |  |  |  |  |
|  | November | 0.002 | 0.003 |  |  |  |  |
|  | December | 0.003 | 0.002 |  |  |  |  |

*Data from 2001

| **Year** | **Month** | **1^st^ half** | **2^nd^ half** | **Year** | **Month** | **1^st^ half** | **2^nd^ half** |
| --- | --- | --- | --- | --- | --- | --- | --- |
| 2003 | January | 0.001 | 0.002 | 2006 | January | 0.001 | 0.001 |
|  | February | 0.002 | 0.001 |  | February | 0.0005 | 0.004 |
|  | March | 0.001 | 0.00003 |  | March | 0.003 | 0.003 |
|  | April | 0.001 | 0.002 |  | April | 0.0004 | 0.002 |
|  | May | 0.002 | 0.003 |  | May | 0.002 | 0.002 |
|  | June | 0.002 | 0.001 |  | June | 0.0003 | 0.002 |
|  | July | 0.002 | 0.002 |  | July | 0.0004 | 0.003 |
|  | August | 0.00001 | 0.003 |  | August | 0.002 | 0.003 |
|  | September | 0.001 | 0.0001 |  | September | 0.001 | 0.001 |
|  | October | 0.002 | 0.002 |  | October | 0.002 | 0.001 |
|  | November | 0.001 | 0.003 |  | November | 0.0003 | 0.003 |
|  | December | 0.001 | 0.002 |  | December | 0.003 | 0.001 |
| 2004 | January | 0.003 | 0.001 | 2007 | January | 0.001 | 0.001 |
|  | February | 0.0001 | 0.0002 |  | February | 0.003 | 0.001 |
|  | March | 0.001 | 0.001 |  | March | 0.002 | 0.002 |
|  | April | 0.001 | 0.002 |  | April | 0.0001 | 0.002 |
|  | May | 0.002 | 0.001 |  | May | 0.001 | 0.004 |
|  | June | 0.0004 | 0.001 |  | June | 0.002 | 0.005 |
|  | July | 0.003 | 0.001 |  | July | 0.005 | 0.003 |
|  | August | 0.003 | 0.002 |  | August | 0.004 | 0.004 |
|  | September | 0.001 | 0.0002 |  | September | 0.0002 | 0.001 |
|  | October | 0.003 | 0.002 |  | October | 0.002 | 0.002 |
|  | November | 0.001 | 0.001 |  | November | 0.001 | 0.001 |
|  | December | 0.0002 | 0.003 |  | December | 0.004 | 0.0004 |
| 2005 | January | 0.001 | 0.002 | 2008 | January* | 0.001 | 0.001 |
|  | February | 0.001 | 0.0004 |  | February* | 0.003 | 0.001 |
|  | March | 0.0003 | 0.002 |  | March* | 0.002 | 0.002 |
|  | April | 0.004 | 0.001 |  | April* | 0.0001 | 0.002 |
|  | May | 0.002 | 0.001 |  | May* | 0.001 | 0.004 |
|  | June | 0.001 | 0.002 |  | June* | 0.002 | 0.005 |
|  | July | 0.002 | 0.001 |  | July* | 0.005 | 0.003 |
|  | August | 0.001 | 0.002 |  | | | |
|  | September | 0.001 | 0.0004 |  |  |  |  |
|  | October | 0.001 | 0.002 |  |  |  |  |
|  | November | 0.001 | 0.001 |  |  |  |  |
|  | December | 0.001 | 0.001 |  |  |  |  |

* Data from 2007.
