# Supplementary material for: Simultaneous Simulations of Uptake in Plants and Leaching to Groundwater of Cadmium and Lead for Arable Land Amended with Compost or Farmyard Manure
Source: PLoS One. 2012 Oct 4;7(10):e47002. doi: 10.1371/journal.pone.0047002 (PMC3464289; doi:10.1371/journal.pone.0047002)
Supplement: Table S3 — Evaporation. Calculated evaporation rates (0.3×ET0) (L m−2 d−1), average half monthly values from August 1998 to July 2008. (DOCX) [file pone.0047002.s003.docx]

**Evaporation**

**Table S3:** Calculated evaporation rates (0.3 x *ET_0_*) (L m^-2^ d^-1^), average half monthly values from August 1998 to July 2008.

| **Year** | **Month** | **1^st^ half** | **2^nd^ half** | **Year** | **Month** | **1^st^ half** | **2^nd^ half** |
| --- | --- | --- | --- | --- | --- | --- | --- |
| 1998 | August | 2.0 | 1.2 | 2001 | January | 0.2 | 0.2 |
|  | September | 0.9 | 1.1 |  | February | 0.3 | 0.4 |
|  | October | 0.4 | 0.5 |  | March | 0.5 | 0.7 |
|  | November | 0.3 | 0.2 |  | April | 0.9 | 0.9 |
|  | December | 0.2 | 0.2 |  | May | 1.0 | 1.7 |
| 1999 | January | 0.2 | 0,2 |  | June | 1.5 | 2.0 |
|  | February | 0.2 | 0.5 |  | July | 1.5 | 1.7 |
|  | March | 0.6 | 0.8 |  | August | 1.8 | 1.5 |
|  | April | 1.0 | 1.1 |  | September | 0.9 | 0.8 |
|  | May | 1.3 | 1.4 |  | October | 0.8 | 0.6 |
|  | June | 1.4 | 1.6 |  | November | 0.3 | 0.2 |
|  | July | 1.7 | 2.0 |  | December | 0.2 | 0.2 |
|  | August | 1.7 | 1.6 | 2002 | January | 0.2 | 0.3 |
|  | September | 1.4 | 0.9 |  | February | 0.4 | 0.4 |
|  | October | 0.7 | 0.6 |  | March | 0.6 | 0.7 |
|  | November | 0.3 | 0.2 |  | April | 1.0 | 1.1 |
|  | December | 0.2 | 0.2 |  | May | 1.0 | 1.3 |
| 2000 | January | 0.2 | 0.2 |  | June | 1.3 | 1.8 |
|  | February | 0.3 | 0.5 |  | July | 1.2 | 1.8 |
|  | March | 0.6 | 0.7 |  | August | 1.2 | 1.1 |
|  | April | 0.9 | 1.0 |  | September* | 0.9 | 0.8 |
|  | May | 1.3 | 1.2 |  | October* | 0.8 | 0.6 |
|  | June | 1.4 | 1.8 |  | November | 0.3 | 0.2 |
|  | July | 1.2 | 1.5 |  | December | 0.1 | 0.1 |
|  | August | 1.8 | 1.4 |  | | | |
|  | September | 1.1 | 1.0 |  |  |  |  |
|  | October | 0.6 | 0.5 |  |  |  |  |
|  | November | 0.3 | 0.2 |  |  |  |  |
|  | December | 0.2 | 0.2 |  |  |  |  |

* Data from 2001.

| **Year** | **Month** | **1^st^ half** | **2^nd^ half** | **Year** | **Month** | **1^st^ half** | **2^nd^ half** |
| --- | --- | --- | --- | --- | --- | --- | --- |
| 2003 | January | 0.1 | 0.2 | 2006 | January | 0.1 | 0.2 |
|  | February | 0.2 | 0.6 |  | February | 0.2 | 0.2 |
|  | March | 0.6 | 1.1 |  | March | 0.4 | 0.5 |
|  | April | 1.0 | 1.3 |  | April | 0.9 | 0.9 |
|  | May | 1.2 | 1.3 |  | May | 1.2 | 1.0 |
|  | June | 1.8 | 1.9 |  | June | 1.8 | 1.6 |
|  | July | 1.8 | 1.6 |  | July | 1.9 | 2.0 |
|  | August | 2.3 | 1.4 |  | August | 1.1 | 1.0 |
|  | September | 1.0 | 1.2 |  | September | 1.2 | 0.9 |
|  | October | 0.6 | 0.4 |  | October | 0.6 | 0.4 |
|  | November | 0.3 | 0.2 |  | November | 0.3 | 0.2 |
|  | December | 0.2 | 0.2 |  | December | 0.2 | 0.1 |
| 2004 | January | 0.2 | 0.2 | 2007 | January | 0.2 | 0.2 |
|  | February | 0.4 | 0.4 |  | February | 0.3 | 0.4 |
|  | March | 0.4 | 0.8 |  | March | 0.7 | 0.5 |
|  | April | 0.8 | 1.1 |  | April | 1.6 | 1.6 |
|  | May | 0.9 | 1.7 |  | May | 1.2 | 1.0 |
|  | June | 1.6 | 1.6 |  | June | 1.7 | 1.2 |
|  | July | 1.2 | 1.7 |  | July | 1.3 | 1.4 |
|  | August | 1.5 | 1.1 |  | August | 1.2 | 1.0 |
|  | September | 1.4 | 0.8 |  | September | 1.0 | 0.8 |
|  | October | 0.5 | 0.5 |  | October | 0.6 | 0.4 |
|  | November | 0.2 | 0.2 |  | November | 0.3 | 0.2 |
|  | December | 0.1 | 0.1 |  | December | 0.2 | 0.2 |
| 2005 | January | 0.2 | 0.2 | 2008 | January* | 0.2 | 0.2 |
|  | February | 0.3 | 0.3 |  | February* | 0.3 | 0.4 |
|  | March | 0.4 | 0.7 |  | March* | 0.7 | 0.5 |
|  | April | 0.8 | 1.0 |  | April* | 1.6 | 1.6 |
|  | May | 1.2 | 1.4 |  | May* | 1.2 | 1.0 |
|  | June | 1.6 | 1.9 |  | June* | 1.7 | 1.2 |
|  | July | 1.5 | 1.4 |  | July* | 1.3 | 1.4 |
|  | August | 1.5 | 1.4 |  | | | |
|  | September | 1.1 | 0.9 |  |  |  |  |
|  | October | 0.8 | 0.6 |  |  |  |  |
|  | November | 0.4 | 0.2 |  |  |  |  |
|  | December | 0.2 | 0.1 |  |  |  |  |

* Data from 2007.
