# Supplementary material for: Simultaneous Simulations of Uptake in Plants and Leaching to Groundwater of Cadmium and Lead for Arable Land Amended with Compost or Farmyard Manure
Source: PLoS One. 2012 Oct 4;7(10):e47002. doi: 10.1371/journal.pone.0047002 (PMC3464289; doi:10.1371/journal.pone.0047002)
Supplement: Table S4 — Plant mass. Estimated grain, leaf, stem and root mass (mg kg fw m−2). (DOCX) [file pone.0047002.s004.docx]

**Plant mass**

**Table S4:** Estimated grain, leaf, stem and root mass (mg kg fw m^-2^)

| **Harvest date** | **Crop** | **Treatment** | **Grain** | **Leaf** | **Stem** | **Root** | **Total** |
| --- | --- | --- | --- | --- | --- | --- | --- |
| 20-10-1999 | Maize | GWS | 2.07 | 1.05 | 3.96 | 2.83 | 9.92 |
| 25-07-2000 | WHEAT | GWS | 1.48 | 0.27 | 2.43 | 2.46 | 6.64 |
| 20-10-2001 | Maize | GWS | 1.85 | 0.99 | 3.72 | 2.65 | 9.22 |
| 20-07-2002 | WHEAT | GWS | 1.36 | 0.20 | 1.82 | 1.84 | 5.22 |
| 15-10-2003 | Maize | GWS | 1.53 | 0.91 | 3.43 | 2.45 | 8.31 |
| 25-07-2004 | WHEAT | GWS | 1.78 | 0.32 | 2.84 | 2.87 | 7.80 |
| 15-10-2005 | Maize | GWS | 1.75 | 0.93 | 3.51 | 2.50 | 8.70 |
| 19-07-2006 | WHEAT | GWS | 1.58 | 0.28 | 2.56 | 2.58 | 7.01 |
| 19-07-2007 | BARLEY | GWS | 1.44 | 0.31 | 2.78 | 2.80 | 7.33 |
| 20-10-1999 | Maize | BIOW | 2.05 | 1.03 | 3.88 | 2.77 | 9.73 |
| 25-07-2000 | WHEAT | BIOW | 1.45 | 0.26 | 2.33 | 2.35 | 6.39 |
| 20-10-2001 | Maize | BIOW | 1.86 | 0.98 | 3.70 | 2.64 | 9.18 |
| 20-07-2002 | WHEAT | BIOW | 1.48 | 0.22 | 2.02 | 2.04 | 5.75 |
| 15-10-2003 | Maize | BIOW | 1.60 | 1.02 | 3.85 | 2.75 | 9.22 |
| 25-07-2004 | WHEAT | BIOW | 1.76 | 0.32 | 2.84 | 2.87 | 7.78 |
| 15-10-2005 | Maize | BIOW | 1.76 | 0.93 | 3.49 | 2.49 | 8.66 |
| 19-07-2006 | WHEAT | BIOW | 1.54 | 0.27 | 2.43 | 2.46 | 6.70 |
| 19-07-2007 | BARLEY | BIOW | 1.38 | 0.23 | 2.09 | 2.11 | 5.82 |
| 20-10-1999 | Maize | FYM | 2.18 | 1.13 | 4.26 | 3.04 | 10.60 |
| 25-07-2000 | WHEAT | FYM | 1.55 | 0.29 | 2.58 | 2.60 | 7.02 |
| 20-10-2001 | Maize | FYM | 1.81 | 0.98 | 3.68 | 2.63 | 9.11 |
| 20-07-2002 | WHEAT | FYM | 1.43 | 0.22 | 2.01 | 2.03 | 5.68 |
| 15-10-2003 | Maize | FYM | 1.67 | 1.00 | 3.75 | 2.68 | 9.09 |
| 25-07-2004 | WHEAT | FYM | 1.76 | 0.32 | 2.88 | 2.91 | 7.87 |
| 15-10-2005 | Maize | FYM | 1.79 | 0.93 | 3.50 | 2.50 | 8.71 |
| 19-07-2006 | WHEAT | FYM | 1.61 | 0.30 | 2.67 | 2.69 | 7.26 |
| 19-07-2007 | BARLEY | FYM | 1.54 | 0.28 | 2.48 | 2.51 | 6.80 |
| 20-10-1999 | Maize | MSW | 2.09 | 1.04 | 3.92 | 2.80 | 9.86 |
| 25-07-2000 | WHEAT | MSW | 1.50 | 0.27 | 2.44 | 2.47 | 6.69 |
| 20-10-2001 | Maize | MSW | 1.97 | 1.01 | 3.81 | 2.72 | 9.52 |
| 20-07-2002 | WHEAT | MSW | 1.56 | 0.24 | 2.12 | 2.14 | 6.05 |
| 15-10-2003 | Maize | MSW | 1.65 | 0.92 | 3.46 | 2.47 | 8.50 |
| 25-07-2004 | WHEAT | MSW | 1.73 | 0.31 | 2.81 | 2.83 | 7.68 |
| 15-10-2005 | Maize | MSW | 1.72 | 0.86 | 3.25 | 2.32 | 8.15 |
| 19-07-2006 | WHEAT | MSW | 1.59 | 0.28 | 2.50 | 2.52 | 6.89 |
| 19-07-2007 | BARLEY | MSW | 1.48 | 0.25 | 2.22 | 2.24 | 6.18 |
| 20-10-1999 | Maize | Control | 2.09 | 1.05 | 3.95 | 2.82 | 9.90 |
| 25-07-2000 | WHEAT | control | 1.44 | 0.27 | 2.40 | 2.42 | 6.52 |
| 20-10-2001 | Maize | Control | 1.80 | 0.95 | 3.59 | 2.56 | 8.91 |
| 20-07-2002 | WHEAT | control | 1.34 | 0.19 | 1.75 | 1.77 | 5.05 |
| 15-10-2003 | Maize | Control | 1.65 | 1.00 | 3.77 | 2.69 | 9.11 |
| 25-07-2004 | WHEAT | control | 1.63 | 0.29 | 2.63 | 2.66 | 7.21 |
| 15-10-2005 | Maize | control | 1.58 | 0.83 | 3.14 | 2.24 | 7.78 |
| 19-07-2006 | WHEAT | Control | 1.45 | 0.26 | 2.32 | 2.35 | 6.38 |
| 19-07-2007 | BARLEY | Control | 1.32 | 0.19 | 1.75 | 1.77 | 5.04 |
