# Supplementary material for: Simultaneous Simulations of Uptake in Plants and Leaching to Groundwater of Cadmium and Lead for Arable Land Amended with Compost or Farmyard Manure
Source: PLoS One. 2012 Oct 4;7(10):e47002. doi: 10.1371/journal.pone.0047002 (PMC3464289; doi:10.1371/journal.pone.0047002)
Supplement: Text S1 — Evaporation. Method for calculating reference evapotranspiration. (DOCX) [file pone.0047002.s005.docx]

**Evaporation**

**Text S1**. Reference evapotranspiration, *ET_0_* (L m^-2^ d^-1^), averaged for 15 days, was calculated from the Penman-Monteith equation (Kay and Davies 2008):

$ET_{0} = \frac{1}{\lambda\rho_{W}} \frac{\Delta\left(R_{n} - G\right) + \frac{\rho_{a} c_{p} \left(e_{a} - e_{d}\right)}{r_{a}} }{\Delta+ \gamma\left(1 + \frac{r_{s} }{r_{a}}\right)} 1000 L m^{-3} 86400 s d^{-1}$ (1)

where

- *R_n_* (J m^-2^ s^-1^) is the net solar radiation. Daily values are given; these are averaged to give one estimate per 15 days,
- *G* (J m^-2^ s^-1^) = 0 is the soil heat flux,
- *ρ_a_* (kg m^-3^) = 1 is the density of air,
- *c_p_* (J kg^-1^ °C^-1^) = 1013 is the specific heat of air,
- *e_a_* (kPa) = 0.611 exp(17.27 *T_a_* / (*T_a_* + 237.3)) is the saturation vapor pressure (*T_a_* (°C) is the air temperature. Daily values are given; these are averaged to give one estimate per 15 days),
- ∆ (kPa °C^-1^) = 17.27 x 237.3 *e_a_* /(*T_a_* + 237.3)^2^ is the slope of the vapor pressure curve,
- *e_d_* (kPa) = *e_a_* x *RH*/100 is the actual vapor pressure (*RH* (%) is the relative humidity. Daily values are given; these are averaged to give one estimate per 15 days)
- *r_a_* (s m^-1^) = 208 / *W* is the aerodynamic resistance (*W* is the wind speed (m s^-1^). Daily values are given; these are averaged to give one estimate per 15 days)
- *λ* (J kg^-1^) = 2.45 x 10^6^ is the latent heat flux
- *ρ_w_* (kg m^-3^) = 1000 is the density of water,
- *γ* (kPa °C^-1^) = 0.066 is the psychrometic constant,
- *r_s_* (s m^-1^) = 70 is the surface resistance of short grass

**REFERENCE**

Kay, A. L. & Davies, H. N. 2008. Calculating potential evaporation from climate model data: A source of uncertainty for hydrological climate change impacts. *Journal of Hydrology*, 358(3-4): 221-239.
