# Supplementary material for: Simultaneous Simulations of Uptake in Plants and Leaching to Groundwater of Cadmium and Lead for Arable Land Amended with Compost or Farmyard Manure
Source: PLoS One. 2012 Oct 4;7(10):e47002. doi: 10.1371/journal.pone.0047002 (PMC3464289; doi:10.1371/journal.pone.0047002)
Supplement: Text S2 — Plant mass. Method for estimating plant mass. (DOCX) [file pone.0047002.s006.docx]

**Plant mass**

**Text S2**. The mass of roots was estimated and added for the total mass of plant, assuming that the dry weight of roots is equal to 31% (maize) and 50% (wheat and barley) of the dry weight of leaves and stems (taken from Yang et al. (2010) for maize and from Rein et al. (2011) for wheat; same percentage assumed for barley and wheat). It was assumed that harvested residues are composed to 79% of stems and to 21% of leaves for maize (http://www.agpm.com/en/mais_plante.php) and to 90% of stems and to 10% of leaves for wheat (Rein et al., 2011); the same proportions as for wheat were also assumed for barley. Fresh weight mass *M* (kg fw) was determined from dry weight mass *M* (kg dw) and water content W (L L^‑1^) as *M* (kg fw) = *M* (kg dw) / (1-*W*) for all plant parts (roots, stems, leaves and grains), and summed up to yield total plant mass on a fresh weight basis. Water contents of roots, stems, leaves and grains were taken as 0.89, 0.8, 0.8 and 0.5 L kg fw^-1^, respectively, for all crops (values taken from Rein et al. (2011) for wheat).

**REFERENCES**

Rein, A., Legind, C. N., & Trapp, S. 2011. New concepts for dynamic plant uptake models. *SAR and QSAR in Environmental Research*, 22(1-2): 191-215.

Yang, C. H., Chai, Q., & Huang, G. B. 2010. Root distribution and yield responses of wheat/maize intercropping to alternate irrigation in the arid areas of northwest China. *Plant Soil and Environment*, 56(6): 253-262.
